# Solitary pulmonary metastasis from primary melanoma of the oesophagus 5 years after resection of the primary tumor

**DOI:** 10.1186/1477-7819-4-22

**Published:** 2006-04-13

**Authors:** Gianlorenzo Dionigi, Francesca Rovera, Luigi Boni, Andrea Imperatori, Renzo Dionigi

**Affiliations:** 1 Department of Surgical Sciences, University of Insubria, Varese, Italy; 2 Center for Thoracic Surgery, University of Insubria, Varese, Italy

## Abstract

**Background:**

Primary malignant melanoma of the oesophagus (PMME) is an uncommon tumor. PMME has an aggressive biological behavior, similar to melanomas developed elsewhere in the body. Most patients die from distant metastases, and the overall 5 year survival rate is approximately 4%.

**Case presentation:**

We report a rare case of a solitary pulmonary metastasis found 5 years after curative resection of primary esophageal melanoma. No other sites of metastatic disease were identified. Video-assisted lung wedge resection of the lung nodule was carried out successfully.

**Conclusion:**

This supports the concept that patients with primary melanoma of the oesophagus treated should be carefully followed up.

## Background

Primary melanoma of the oesophagus (PMME) is an uncommon tumor with an incidence of 0.1% of esophageal cancer and represents about 0.5% of noncutaneous melanomas [1]. Firstly Baur reported a case in 1906 [2]. PMME was not widely accepted until de la Pava in 1963 demostrated the presence of melanocytes in normal oesophageal mucosa in 4 of 100 normal esophagus at autopsy examination. Tateshi reported an incidence of 8% for the presence of melanocytes in the stratum basale of normal esophagi of Japanese subjects [4]. Although the presence of normal melanocytes in the esophagus has been largely demonstrated, their origin is still debatable [3]. Suzuki in a large study on surgical and autopsied specimens reported a 0.1% and 0.14% incidence respectively [5]. Two-hundreds cases are described in literature. Almost 90% of PMME are located in the lower third of the esophagus and melanosis seems to be a predisposing factor since it as been found in almost 25% of the patients suffering for PMME [6]. Diagnosis is made by exclusion criteria with no other primary cutaneous, ocular, mucosal lesion detected. Histologically, the tumor is considered as primary when it presents with a characteristic structure of melanoma and contains pigment (melanin), neighboring melanosis or melanocytic dysplasia is generally required to distinguish it from metastatic disease, the tumor is often polypoid and arise in an area of junctional changes in the squamous epithelium [7]. Histopathological examination is usually mandatory to reach a definitive diagnosis since symptoms are not different from those of other malignant tumors of the esophagus. It is associated with a poor survival, also when resected at an early stage. In fact the tumor has an aggressive biological behavior, similar to melanomas occurred elsewhere in the body. Most patients die from distant metastases, and the 5 year survival rate is approximately 4%. At the time of presentation, 40% of patients have already metastatized, primarily to regional lymph nodes, liver, lung, or bones [8]. Although pulmonary metastasis from melanoma is not uncommon, the case we report of a solitary pulmonary metastasis found 5 years after curative resection of the primary esophageal tumor is extremely unusual [9,10]. A review of recent literature is also presented.

## Case presentation

**Figure 1:**
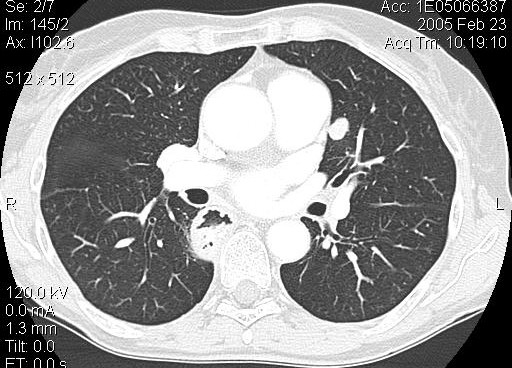
Computed tomography: well delimited pulmonary nodule of 13 mm in diameter of the LUL.

**Figure 2:**
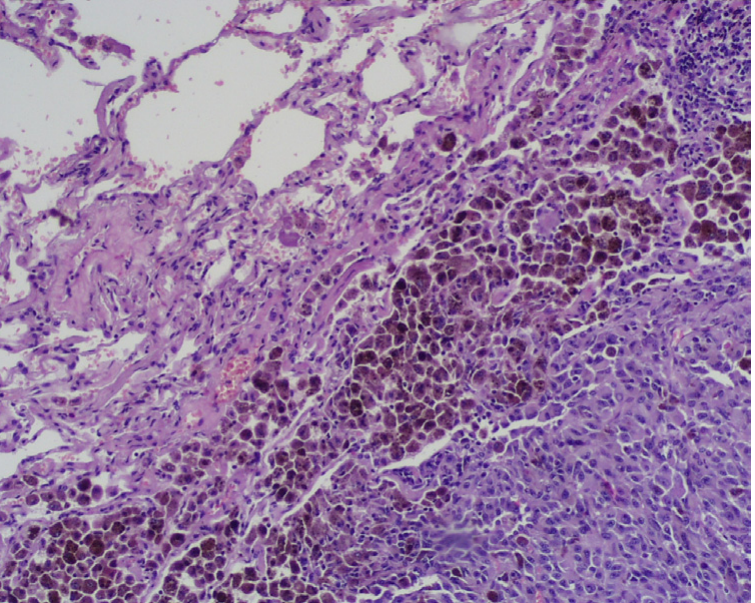
Histology: section through pulmonary tissue showing metastatic melanoma (hematoxylin and eosin x40 and x100).

**Figure 3:**
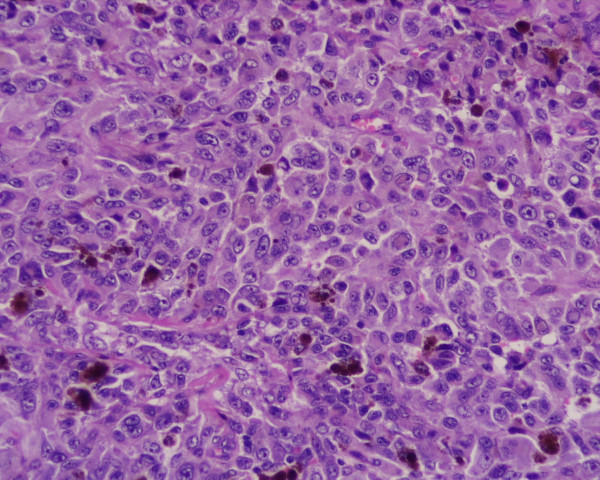
The tumor cells are intesively positive for immuno-reaction, using HMB45 anti-melanoma antibodies.

A 62-year-old caucasian woman, who had undergone curative resection of primary melanoma of the oesophagus 5 years earlier without adjuvant chemo-radiotherapy [9,10], was referred to our hospital for a solitary pulmonary nodule (13 × 10 mm) in the left upper lobe (LUL) detected during routine follow-up chest X-ray examination. Thoracic computed tomography (CT) showed a well delimited round tumor, about 13 mm in diameter located in the LUL, with no mediastinal lymph-nodes enlargement (Figure 1); brain and abdominal CT performed for staging did not find distant metasteses. Staging was then compleated with flexible endoscopy (the remnant esophagus, stomach and duodenum were normal), immunoscintigraphy with 99 mTc-labeled melanoma monoclonal, bone scan and broncoscopy which resulted all negative. Laboratory tests were unremarcable. Video-assisted lung wedge resection of the pulmonary nodule was carried out successfully. A definitive pathological diagnosis was achieved: the macroscopic examination of the surgical specimen confirmed the CT findings. The final histology confirmed proliferation of small spindle-shaped or stellate cells arranged in a spiral or fascicular structure, the tumor cells were intesively positive for immuno-reaction, using HMB45 anti-melanoma antibodies (Figure 2, 3). These findings were compatible with the diagnosis of metastases of primary esophageal melanoma. The patient had an uneventful postoperative course and is still well without any evidence of further recurrence 6 months after surgery. This is a rare case of solitary lung metastasis appearing 5 years after eradication of primary esophageal melanoma.

## Discussion

Several explanations have been postulated for delayed metastasis of differentiated tumors. Alexander presented an intriguing pathogenic hypothesis proposing that clusters of dividing cells lead to an equilibrium between cell death and proliferation, or that the tumor cells remain in a state of rest for long periods without losing their viability before renewed tumor growth is induced by the somatic transformation of the cancer cells [11]. We report a rare case of a solitary pulmonary metastasis found 5 years after curative resection of primary esophageal melanoma. Radical surgical resection with *en bloc *lymphadenectomy is the treatment of choice of primary melanoma of the oesophagus, with a five year survival of 4.25% [8]. Although laser, radiotherapy, chemotherapy, immunotherapy have not proven to be beneficial, they may play a palliative role if surgery is not applicable for advanced stages or poor functional status [12]. Of the approximately 200 cases that have been reported to date, only about 30% of the patients survived more than 1 year after the initial diagnosis [8]. The aggressive biological behavior of this disease, the advanced stage at the time of diagnosis and the lack of effective therapy contribute to its poor prognosis. PMME is a very aggressive tumor and esophagogastroduodenoscopy, endoscopic ultrasonography and CT scan are required to complete the preoperative staging. Chalkiadakis demonstrated that distant metastases are present in 78% of the patients suffering from PMME [13]. The most common site of metastases is the liver (31%), followed by mediastinum (29%), lung (17%), brain (13%) and other intra-abdominal organs. Detection rate of metastatic disease identification using immunoscintigraphy with 99 mTc-labeled melanoma monoclonal AB is 95% for bone lesions, 91% for liver, 78% for lymph node metastases, 62% for brain, 62% for spleen and 58% for lung [14,15]. This technique cannot identify deposits of 1 cm or less in size [14,15]. Although it has been demonstrated that radical resection increases the survival compared to local treatment, 5-year survival is less than 5%, mainly due to the advanced state of the disease at the time of diagnosis [16]. Various drugs, alone or in combination, including vinblastine, bleomycin, lomustine, vincristine and recently recombinant interferon-alpha and interleukin-2, have been used with varying results as palliative treatment of cutaneous melanoma [17-20]. Combination of cisplatin, carmustine, dacarbazine and tamoxifen have also been studied with promising results in cutaneous melanoma patients with advanced disease [21]. Several investigators have supported the above findings and the role of tamoxifene as drugs modulator [22-24]. Nevertherless, none of these regimens have been used in esophageal melanomas which is considered to be a radioresistant tumor, and chemotherapy, external beam and intracavitary radiotherapy may only have a palliative role [25]. At the time of diagnosis, clinically, surgically or pathologically detectable metastases were present in 40.9% of patients. The overall survival is 9.8 months with five year survival rate of 1.69%. Only 33% of patients survive for more than 1 year after diagnosis. Deaths are disease-related in 75–85% of cases [8].

Although the reason for the delayed presentation of the metastatic lesion remains unclear, the case report we described demonstrates that patients with primary melanoma of the oesophagus surgically treated should be carefully followed up.

## Competing interests

The author(s) declare that they have no competing interests.

## Authors' contributions

**GD: **acquisition of data, **FR: **study conception and design, **LB**: analysis and interpretation of data, **GD, AI: **drafting of manuscript, **RD: **Critical revision and supervision
